# Effect of differences in light source environment on transcriptome of leaf lettuce (*Lactuca sativa* L.) to optimize cultivation conditions

**DOI:** 10.1371/journal.pone.0265994

**Published:** 2022-03-29

**Authors:** Soichiro Nagano, Naoya Mori, Yukiko Tomari, Noriko Mitsugi, Ayumi Deguchi, Makoto Kashima, Ayumi Tezuka, Atsushi J. Nagano, Hitohide Usami, Takanari Tanabata, Hiroyuki Watanabe

**Affiliations:** 1 Department of Advanced Food Sciences, Faculty of Agriculture, Tamagawa University, Machida, Tokyo, Japan; 2 Department of Frontier Research and Development, Kazusa DNA Research Institute, Kisarazu, Chiba, Japan; 3 Tamagawa University Research Institute, Machida, Tokyo, Japan; 4 Research Institute for Food and Agriculture, Ryukoku University, Otsu, Shiga, Japan; 5 Faculty of Agriculture, Ryukoku University, Otsu, Shiga, Japan; 6 Institute for Advanced Biosciences, Keio University, Tsuruoka, Yamagata, Japan; Universidade de Lisboa Instituto Superior de Agronomia, PORTUGAL

## Abstract

When used in closed-type plant factories, light-emitting diode (LED) illumination systems have the particular advantages of low heat emission and high luminous efficiency. The effects of illumination quality and intensity on the growth and morphogenesis of many plant species have been examined, but improvements are needed to optimize the illumination systems for better plant products with lower resource investments. In particular, new strategies are needed to reduce the wastage of plant products related to leaf senescence, and to better control the ingredients and appearance of leafy vegetables. Although the quality of light is often altered to change the characteristics of plant products, the transcriptional status underlying the physiological responses of plants to light has not been established. Herein, we performed a comprehensive gene expression analysis using RNA-sequencing to determine how red, blue, and red/blue LEDs and fluorescent light sources affect transcriptome involved in the leaf aging of leaf lettuce. The RNA-sequencing profiling revealed clear differences in the transcriptome between young and old leaves. Red LED light caused large variation between the two age classes, while a pure or mixed blue LED light spectrum induced fewer transcriptome differences between young and old leaves. Collectively, the expression levels of genes that showed homology with those of other model organisms provide a detailed physiological overview, incorporating such characteristics as the senescence, nutrient deficiency, and anthocyanin synthesis of the leaf lettuce plants. Our findings suggest that transcriptome profiles of leaf lettuce grown under different light sources provide helpful information to achieve better growth conditions for marketable and efficient green-vegetable production, with improved wastage control and efficient nutrient inputs.

## Introduction

A plant factory with artificial illuminating devices is an enclosed cultivation system that enables the control of environmental factors such as illumination, temperature, humidity, CO_2_ concentration, and nutrient conditions. Such closed types of plant factories are advantageous for urban vegetable gardens, providing a safe supply of food products with stable yields and quality in the absence of pesticides and without using excess water or nutrients. Such plant factories have been used commercially for production of leafy vegetables in Japan, China, and Taiwan [1].

The light spectral and economic characteristics of a variety of light sources for plant production have been evaluated, including high-voltage sodium lamps, metal-halide lamps, fluorescent lamps, hybrid electrode fluorescent lamps, and light-emitting diodes (LEDs), and these light sources have been used for better plant cultivation. Recently, LED illumination systems have been widely adopted in closed-type plant factories; these systems offer the advantages of lower heat emission, longer operating lifespan, and narrower spectral output [2].

The effects of the quality and intensity of the light on the growth and morphogenesis of many plant species have been examined. For example, despite their different photosynthetic photon flux densities, lettuce plants (*Lactuca sativa* L.) grown under red LEDs have been shown to develop more leaves than the plants grown under blue LEDs [3], whereas pepper plants (*Capsicum annuum* L.) showed a reduced number of leaves when grown under red LEDs [2]. Compared to plants grown under red LEDs with supplemental blue LEDs or cool white fluorescent light, plants grown under only red LEDs exhibited lower dry mass accumulation in radish (*Raphanus sativus* L.), spinach (*Spinacia oleracea* L.), and lettuce [4]. Pepper [2] and wheat (*Triticum aestivum* L. [5]) also have shown increased dry mass under red LEDs with supplemental blue light in comparison with red LEDs. These results suggested that the optimal light quality for plant growth differs among plant species [6].

Several further improvements are needed to optimize illumination systems for better plant products with lower investments of resources. Two of the most important tasks are to (1) diminish the proportion of commercially undesirable plant products (e.g., senescent leaves), and (2) control the yield and ingredients in leafy vegetables. Because senescent leaves turn yellow and wilt, the market price of vegetables with senescent leaves is lower, and thus such leaves must be manually removed by laborers. The development of cultivation methods that delay leaf senescence has been another challenge in the efforts to improve product yields and the profitability of plant factories [7].

Leaf senescence is the final stage of leaf development, and it is an important process for the relocation of nutrients from the senescent leaves to new organs. Many environmental factors influence leaf senescence, e.g., drought, nutrient limitation, extreme temperature, oxidative stress by UV-B irradiation, and ozone; these are summarized by Lim et al. [8] and Ono and Nagano [9]. The effect of changes in light quality on leaf senescence have been studied in many species; these are reviewed by Causin and Barneix [10], and Guo et al. [11]. Leaf senescence occurs at low light intensity in the cases of self and mutual shading, with a higher light-attenuation rate in the upper leaves [12]. In addition, hormonal signaling pathways influence developmental and environmental responses in leaf senescence as internal factors. For example, an accumulation of cytokinins retards leaf senescence [13], and exogenous abscisic acid promotes leaf senescence [14]. These environmental and internal factors are interconnected to form a complex regulatory network [15, 16].

In a plant factory, plants growing under their own shade and/or shade from neighboring plants are the most likely to undergo leaf senescence, because the shaded lower leaves senesce faster than upper leaves. Zhang et al. examined the effects of supplemental upward illumination with white, red, and blue LEDs on the abaxial side of lettuce leaves from underneath, and they reported that supplemental illumination both retarded the leaf senescence of outer leaves and decreased wastage of cultivation products [7]. To improve cultivation methods for leaf lettuce, it is necessary to clarify the mechanisms underlying the different effects of light sources on the physiological regulation involved in leaf aging.

Large numbers of senescence-associated genes (SAGs) have been identified in *Arabidopsis thaliana* by microarray [17] and RNA-sequencing (RNA-Seq) [18] analyses. Investigations have unveiled the red and blue light regulatory responses in transcriptomes in *Arabidopsis thaliana* [19], seaweed (*Saccharina japonica* [20]), and Norway spruce [21]. However, there have been few comprehensive gene expression analyses of leafy vegetables in relation to the detection of fungal responses [22], circadian oscillation [23], and the effects of nanoparticle treatment [24] in lettuce and in cultivated and wild spinach [25]. Zang et al. published the transcriptome profile of leaf lettuce in their efforts to determine the effects of white-light intensity on the anthocyanin synthesis pathway [26]. Kitazaki et al. evaluated the transcriptional and metabolic responses to different light qualities and intensities in leaf lettuce seedlings grown under narrow-band LED illumination, and also examined the effects of different types of light on the balance between biomass production and secondary metabolites [27]. To the best of our knowledge, no studies have examined the effect of different types of light on transcriptome in relation to leaf senescence.

In the present study, we performed a comprehensive gene expression analysis using RNA-Seq to explore how different light sources affect transcriptome involved in leaf aging in leaf lettuce. Transcriptome data provide useful information in lettuce cultivation management, as these data can be expected to reflect the physiological conditions of the plant. Based on our present findings, we discuss transcriptome profiles of young and old leaves of lettuce grown under different light sources in our ongoing effort to identify the optimal growth conditions including illumination for green-vegetable production.

## Materials and methods

### Plant materials and growth conditions in a plant factory with artificial illumination

Seeds of a cultivar of leaf lettuce (*Lactuca sativa* L.), ’Red fire’ (Takii Seed Co., Kyoto, Japan) were sown on sponge blocks (2.3 cm wide, 2.3 cm deep, 2.7 cm high) soaked with water. After germination, the seedlings were grown at 23.0 ± 2.0°C for 14 days under 16 h/8 h light/dark with 100 ± 20 μmol photons m^−2^ s^−1^ fluorescent light. On the 19th day after the seed sowing, the seedlings were transplanted into cultivation compartments with four different types of light sources: (1) red LEDs (R), (2) blue LEDs (B), (3) a mixture of the red and blue LEDs (RB, R:B = 7:3), and (4) fluorescent light (FL). The light characteristics are listed in Table 1, and the spectrum is illustrated in S1 Fig. The light intensities were adjusted to 150 ± 20 μmol photons m^−2^ s^−1^ at the plant height. Hydroponic systems were used for plant growth with Otsuka type-A nutrient solution (OAT Agrio Co., Tokyo, Japan). The EC and pH of the hydroponic solution were maintained at 1.5 ± 0.1 dS m^−1^ and 6.0 ± 0.5, respectively. The air temperature was maintained at 23.0 ± 2.0°C, and the relative humidity was kept at 65 ± 10%. The minimum CO_2_ concentration was maintained above 1,000 μmol mol^−1^.

**Table 1:** Growth conditions of the plant materials.

Light source	Abbreviation	Peak wavelength, nm	Intensity, μmol m^-2^s^-1^	Duration light / dark, hr	Temperature, ˚C	Relative humidity, %	Min CO_2_ concentration, μmol mol^-1^	Hydroponic solution	
								EC, dS m^-1^	pH
For seedling growth									
Fluorescent	FL	405, 436, 489, 545, 586, 612, 709	100 ± 20	16 / 8	23.0 ± 2.0	65 ± 10	1,000	1.0 ± 0.1	6.0 ± 0.5
For plant cultivation									
Red	R	659	150 ± 20	24 / 0	23.0 ± 2.0	65 ± 10	1,000	1.5 ± 0.1	6.0 ± 0.5
Blue	B	443	150 ± 20	24 / 0	23.0 ± 2.0	65 ± 10	1,000	1.5 ± 0.1	6.0 ± 0.5
Red/Blue	RB	443, 659	150 ± 20	24 / 0	23.0 ± 2.0	65 ± 10	1,000	1.5 ± 0.1	6.0 ± 0.5
Fluorescent	FL	405, 436, 489, 545, 586, 612, 709	150 ± 20	24 / 0	23.0 ± 2.0	65 ± 10	1,000	1.5 ± 0.1	6.0 ± 0.5

EC: electrical conductivity.

On the 21st day after transplantation into the growth compartments, the plants were harvested for fresh mass and transcriptome analyses. Under all four light conditions, an old (O) leaf was defined as the eighth leaf from the oldest leaf in an individual plant that had been fully expanded for >5 days. Leaves that were visibly showing senescence were not included in the samples. Since there were differences in the number of expanded leaves among the plants grown under the different light sources (Table 2), we used young (Y) leaves >10 cm long that continued expanding in an individual plant as follows: the 16th or 17th leaf (from the oldest leaf) for the red LEDs, the 13th or 14th leaf for the blue LEDs, the 14th to 16th leaf for the red/blue LEDs, and the 16th or 17th leaf from the oldest leaf for the fluorescent light. Here, we avoided sampling individuals that had wilting on three leaves before and after the target leaf. For the transcriptome analysis, 8-mm-diameter leaf discs of each sample were taken with a cork borer, put into a microtube, and frozen with liquid nitrogen. The microtubes were stored at −80°C.

**Table 2:** Phenotypic characteristics in the plants grown under different light conditions.

Light source	Abbreviation	Above-ground fresh mass, g plant^-1^	Above-ground dry mass, g plant^-1^	Below-ground dry mass, g plant^-1^	Number of leaves, plant^-1^
Red	R	213.7 ± 16.3 ^a^	9.4 ± 1.4 ^a^	1.1 ± 0.2 ^a^	25 ± 2 ^b^
Blue	B	158.8 ± 19.4 ^b^	8.2 ± 1.3 ^a^	0.8 ± 0.2 ^a^	22 ± 2 ^b^
Red/Blue	RB	157.0 ± 10.7 ^b^	7.7 ± 1.0 ^a^	1.1 ± 0.2 ^a^	24 ± 2 ^b^
Fluorescent	FL	219.3 ± 49.5 ^a^	10.3 ± 1.9 ^a^	0.8 ± 0.2 ^a^	29 ± 2 ^a^

Different superscripts indicate significant differences (p<0.05) with ANOVA and Tukey’s HSD post-hoc test (n = 4–5 replicate samples).

### Analysis of phenotypic characteristics

After 21 days from their transplantation to the growth compartments, the individual plants’ fresh mass values were determined for five plants for each type of light. Plants were removed from the hydroponic equipment, and droplets of water were wiped from their roots before the plants were weighed with a balance. After the fresh mass evaluation, the number of leaves on each plant was counted. The plants were separated into above- and below-ground parts, and weighed. The effects of the light source on the phenotypic characteristics were statistically analyzed with analysis of variance (ANOVA), and homogeneous groups were determined by Tukey’s honestly significant difference (HSD) post-hoc test by using a software JMP (SAS Institute Japan Inc., Tokyo, Japan).

### Anthocyanin measurements

The leaf blades of young and old leaves were used for anthocyanin measurements: each 1.0 g fresh weight sample was homogenized with a mortar and pestle in 9.0 mL of 1% hydrochloric acid in methanol. Anthocyanin in the homogenate was extracted overnight in the dark at 4°C. The homogenate was centrifuged at 3,000 g for 15 min. The absorbance of the supernatant was then measured at a wavelength of 530 nm using a spectrophotometer (UV-1800; Shimadzu Co. Ltd., Kyoto, Japan) and standardized with cianidin-3-glucoside to determine the anthocyanin content.

### Transcriptome analysis

Total RNA was extracted with an RNA Extraction Plant Mini kit (Qiagen, Hilden, Germany), and the integrity of the extracted RNA was evaluated with a Bioanalyzer 2100 and an RNA 6000 Nano kit (Agilent Technologies, Santa Clara, CA, USA). The mRNA purification and library preparation were conducted as described in our previous study [28] with an automated liquid handling system (Freedom EVO 150; Tecan, Zürich, Switzerland) and a thermal cycler (ODTC 384; Inheco, Munich, Germany). The quality of the RNA-Seq library was evaluated with a Bioanalyzer 2100 and High Sensitivity DNA kit (Agilent Technologies). Sequencing for 50 bp single-end reads was carried out by Macrogen Japan Co. (Kyoto, Japan) using a HiSeq2500 (Illumina, San Diego, CA, USA). The sequence data were deposited to the DDBJ Sequence Read Archive under accession number DRA008960.

All obtained reads were trimmed with the tool Trimmomatic (ver. 0.3.3, [29]), using the following parameters: TOPHRED33 ILLUMINACLIP: TruSeq3-SE.fa:2:30:10 LEADING:19 TRAILING:19 SLIDINGWINDOW:30:20 AVGQUAL:20 MINLEN:40. This procedure removed adapter sequences (ILLUMINACLIP:TruSeq3-PE.fa:2:30:10) and leading and trailing low-quality or N bases (below quality 19) (LEADING:19 TRAILING:19). In addition, the trimming of reads from the 3’ end was conducted when the average quality per base was below 20 with a 30-base-wide sliding window (SLIDINGWINDOW:30:20).

Finally, trimmed reads >39 nucleotides long with an average quality score >19 were output. The trimmed reads were then mapped on the reference UniGene of lettuce on the U.S. National Center for Biotechnology Information (NCBI) repository (ftp://ftp.ncbi.nih.gov/repository/UniGene/Lactuca_sativa/Lsa.seq.uniq.gz) with RSEM (ver. 1.3.0, [30]), using Bowtie (ver. 1.1.2, [31]) with default parameters. The raw counts of all samples were combined into a file and treated with the trimmed mean of M-values (TMM) normalization using edgeR package (ver. 3.18.1, [32]) in R software, (ver. 3.3.2, [33]). The reference annotation of the lettuce gene was subjected to a reciprocal BLAST search against TAIR10, a database for *Arabidopsis thaliana* (http://www.arabidopsis.org/index.jsp). Of the total of 45,536 lettuce genes, we used 25,047 genes (11,913 genes were reciprocally annotated and 13,134 genes showed one-side hits) for the examination of functions.

We performed a clustering analysis and principal component analysis (PCA) with the TMM-normalized counts to determine the similarity of the transcriptome for all of the samples. Spearman’s rank correlation coefficients were calculated with all of the transcriptome data of the 40 samples. The coefficients were divided from 1 and were defined as the distance within a cluster dendrogram. We used the top 200 genes with the largest variance (S2 Table) to calculate the principal components. The cluster dendrogram and PCA plots were generated with the R software. The expression levels of the 200 genes with the largest variance were also plotted as a heat map with the R software.

We detected differentially expressed genes (DEGs) by comparing the young leaves and old leaves for each light source condition, and we set cutoffs based on the results of the exact tests using the edgeR package [32] to evaluate the differences in means between two groups of negative binomial random variables. The numbers of DEGs were recorded for each light source condition with false discovery rates (FDRs) of <10^−5^, 10^−4^, and 10^−3^. The relationships of the log fold change (M) and the log of an average expression level (A) between the young and old leaves are presented as an MA plot for each of the four light source condition with the edgeR package. The similarity and specificity of the differentially expressed genes are presented as a four-set Venn diagram. The list of the commonly detected genes in the comparison of the young and old leaves through the four light conditions is provided as S3 Table.

The gene expression levels related to leaf senescence [17, 18, 33–38] and those related to the anthocyanin synthesis pathway [39] were extracted as tables with their annotations and are expressed herein as bar plots and heat maps, respectively. For the genes related to leaf senescence, we analyzed the expression levels by performing an ANOVA with Tukey’s HSD test with the R software.

We performed gene ontological enrichment analyses with BinGO (ver. 3.0.3, [40]), a plug-in for Cytoscape (ver. 3.6.0, [41]). The GO annotations are attached for the DEGs detected by the exact test in edgeR [32] in our comparison of transcriptomes of the young leaves grown under the red LED and the blue LED light conditions. A GO annotation file for *Arabidopsis thaliana* built into the BinGO software was used for the enrichment analysis. We observed that 11,913 lettuce genes that were reciprocally annotated were filtered from the extracted DEGs. GO pathways of three categories, i.e., Biological Process (BP), Cellular Component (CC), and Molecular Function (MF), were drawn for upregulated genes under the red LED light condition and the blue LED light condition.

### Gene expression analysis with real-time RT-PCR

To validate the expression levels of genes related to leaf senescence and to anthocyanin metabolic pathway, we performed real-time reverse transcription-polymerase chain reaction (RT-PCR) analysis, using the leaf blades of young and old leaves. The sample was homogenized with liquid nitrogen using a mortar and pestle. Approximately 50 mg of the powdered sample was transferred into a tube, and 1 mL of Fruit-mate (Takara Bio, Shiga, Japan) was added before vortexing the tube. The tube was then centrifuged for 5 min at 14,000 rpm and 4°C, and the resulting supernatant was transferred to a separate tube. Total RNA was extracted using a Maxwell RSC Plant RNA Kit (Promega, Madison, WI, USA) and a Maxwell RSC Instrument (Promega). A tube containing total RNA and PrimeScript RT reagent Kit (Takara Bio) was heated in a block incubator for 15 min at 37°C, then for 5 s at 85°C, before cooling down to 4°C. Then, 2 μL of the reaction solution, 12.5 μL of TB Green Premix EX Taq II (Takara Bio), 1 μL each of the forward and reverse primers, and 8.5 μL of sterilized purified water were mixed in an optical microtube. The relative gene expression levels were quantified using a Thermal Cycler Dice Real Time System TP970 (Takara Bio).

The expression levels of the genes, *REV*, *WRKY75*, *PAL*, *CHS*, and *ACT7* were analyzed. The sequences of each primer used in the analysis are shown on S4 Table. The relative expression level of each gene was calculated with *ACT7* [42] as the control gene and with average of the young leaves grown under the red LED light conditions as the control sample using the 2^-ΔΔCT^ method [43]. The effect of light source conditions on the relative expression levels of five replicate samples were analyzed with ANOVA, and homogeneous groups were determined by Tukey’s HSD post-hoc test using the R software.

## Results

### Plant phenotypic characteristics of the leaf lettuce grown under different light conditions

The above-ground fresh mass values of the plants grown under the red LEDs and those grown under fluorescent light were significantly greater than those of the plants grown under the blue LEDs and red/blue LEDs (Table 2). The above-ground dry mass of the plants grown under the fluorescent light was also significantly greater than those of the plants grown under the blue LEDs or red/blue LEDs (Table 2).

The number of leaves in the plants grown under the fluorescent light was greater than that of the plants grown under the other light conditions (Table 2). The root dry mass values of the plants grown under the red LEDs and the red/blue LEDs were significantly greater than those of the plants grown under the blue LEDs or fluorescent light sources (Table 2). These results suggest that red light increases the plant mass of leaf lettuce, whereas blue light decelerates the plant growth.

**Fig 1:**
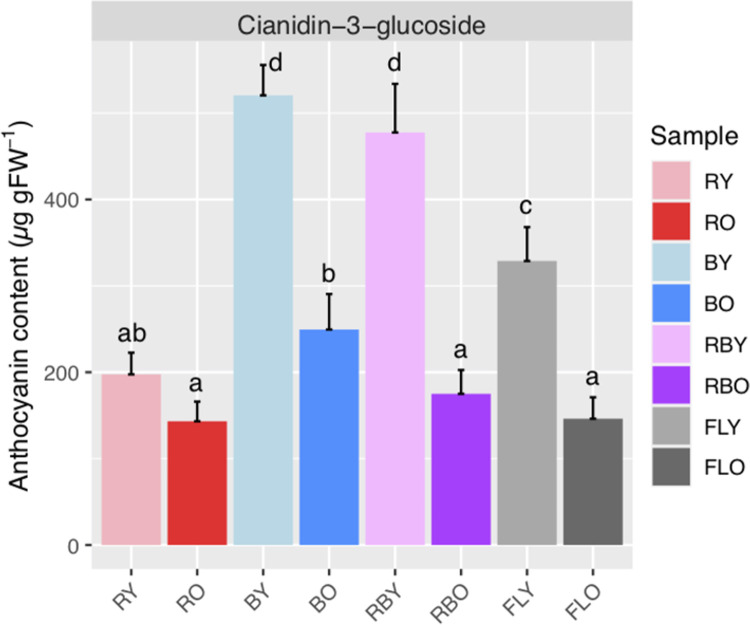
Anthocyanin content in the young (Y) and old (O) lettuce leaves grown under the red (R), blue (B), and red/blue (RB) LEDs and the fluorescent (FL) light source conditions. Mean ± SD values for 5 replicate samples are shown. Different superscripts on the bars indicate a significant difference (p<0.05) by ANOVA with Tukey’s HSD test.

The fresh-weight based anthocyanin content tended to be decreased with leaf aging under all four light conditions (Fig 1). The young leaves grown under the red LEDs had significantly lower anthocyanin contents than those grown under the blue LEDs, red/blue LEDs, and fluorescent light sources, whereas there was no significant difference in the anthocyanin content in the old leaves grown under the red LEDs, red/blue LEDs, and fluorescent light sources (Fig 1).

### Summary statistics of the transcriptomes

The summary statistics of the transcriptomes are given in S1 Table. An average of 5.2 ± 2.7 million reads from 1.1 to 17 million reads per sample were acquired for the total of 40 samples (S1 Table) with two lanes of the single-end 50 cycles in the HiSeq2500 run. The averaged Q score of the reads was 36.9 throughout the 40 samples. The averaged mapped rate was 90.0 ± 2.8% on the UniGene of lettuce.

### Similarity of the transcriptomes among the different light conditions

**Fig 2:**
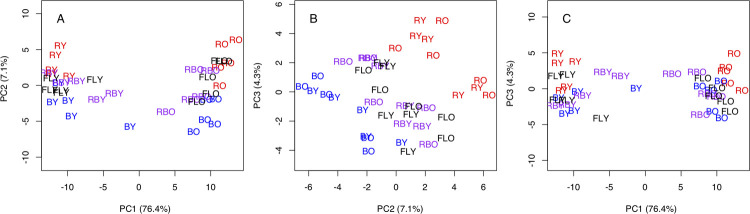
Scatter plots of the PCA scores derived from the leaf transcriptome in the plants grown under the red (R), blue (B), and red/blue (RB) LEDs and the fluorescent (FL) light source conditions. The leaf samples were harvested from young (Y) and old (O) leaves of each of five individual plants grown under the different light sources. The relationships between PC1 and PC2 (A), between PC2 and PC3 (B), and between PC1 and the PC3 (C) are presented with the respective variance proportions.

Our rank correlation coefficient analysis with all of the transcriptome data produced a cluster dendrogram that placed the young leaves and the old leaves in different large nodes (S2 Fig). In the PCA, the transcriptome profiles of the young and old leaves fit along two main axes. The proportions of variance for the principal components are shown in Fig 2A. The first axis (PC1, 76.4%) was the most highly correlated with leaf aging (Fig 2A). The largest difference in PC1 was observed between the young and old leaves of the plants grown under the red LEDs, whereas the differences in PC1 were small between the young and old leaves of the plants grown under the blue LEDs and the red/blue LEDs (Fig 2A).

The second axis (PC2, 7.1%) presented differences between the light sources and the variability of the spectral composition of the light. Close proximity of the light sources within the age categories reflects the simplicity of the spectral compositions in the red LEDs and the blue LEDs, except for several samples. The young and old leaves of the plants grown under the red LEDs were tightly grouped in the relationship between PC1 and PC2, although the difference along the PC1 was the largest in the comparison of all of the light conditions (Fig 2A). The plants grown under the blue LEDs and those grown under the red/blue LEDs showed a wide distribution of young and old leaves. For the plants grown under fluorescent light, the young and the old leaves were grouped within red LED and blue LED light samples in each leaf-age category. Although the contribution of the third axis (PC3, 4.3%) was small compared to those of PC1 and PC2, PC3 was the most highly correlated with the light source conditions (Fig 2B and 2C).

### Genes differentially expressed between young and old leaves

**Fig 3:**
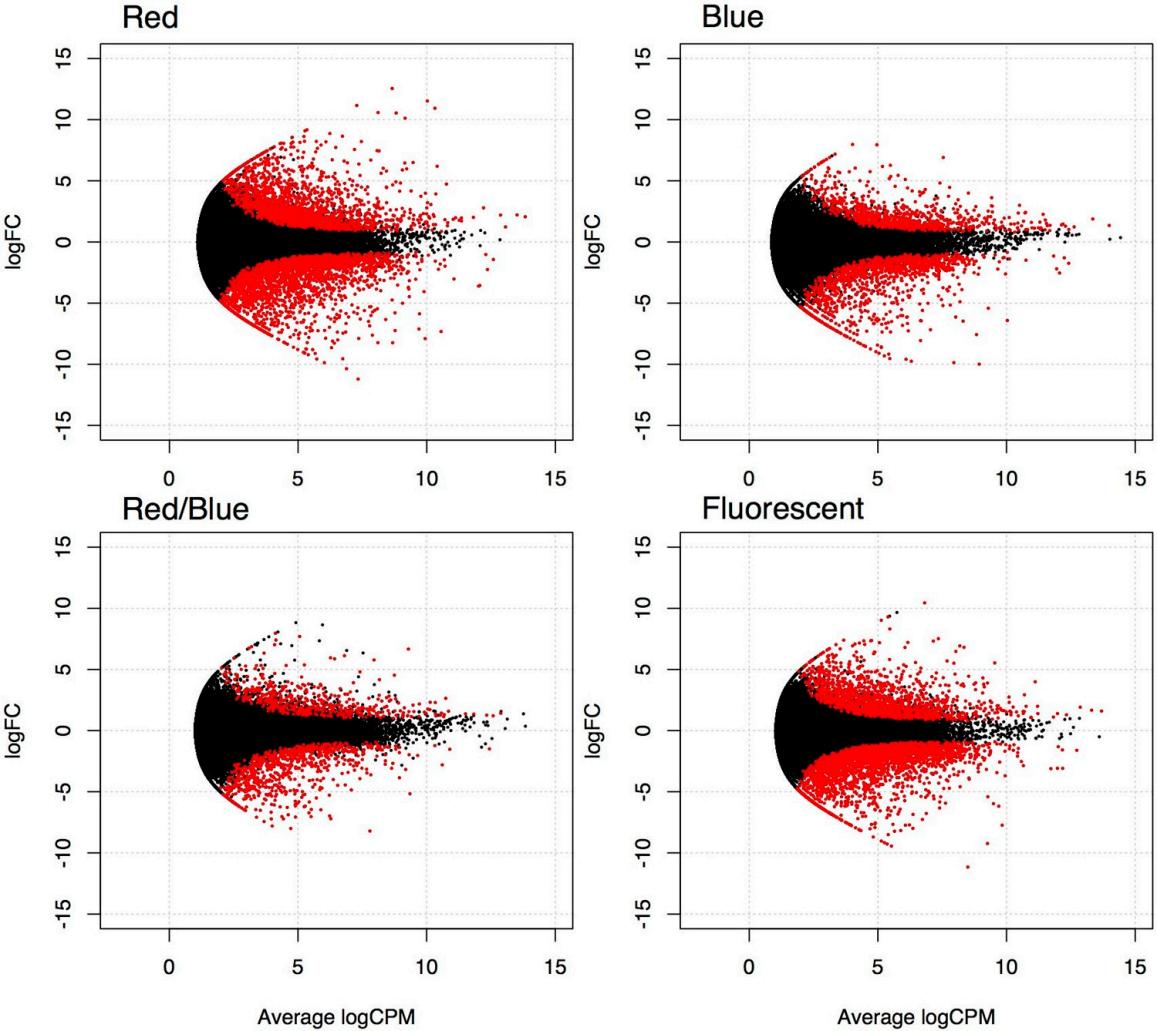
MA plots for different light source conditions. Relationships of the log_2_ fold change (FC) and the average of log_2_ counts per million (CPM) between the young and old leaves of lettuce plants grown under the four light source conditions. Genes differentially expressed between the developmental stages (FDR<1*10^−3^) are highlighted in red.

Our comparison of the young and old leaves of plants grown under the different light conditions revealed that the number and composition of the DEGs depended on the light sources. The highest number of DEGs between young and old leaves (i.e., 4,257) was observed under the red LED condition when FDR = 0.001 was used as the significance level (Fig 3 and Table 3), although the number decreased when the significance level was tightened to FDR<0.00001 or FDR<0.0001 (Table 3). The second-highest number of DEGs (i.e., 4,097 at FDR < 0.001) was observed under the fluorescent light tubes, and the number was smaller for the plants grown under the blue LEDs (1,968 genes) or red/blue LEDs (1,247 genes) (Fig 3 and Table 3). In the comparison of the genes up-regulated and down-regulated in old leaves, the number of down-regulated genes was higher than that of the up-regulated genes under all light conditions (Table 3).

**Table 3:** Number of differentially expressed genes between the young and the old leaves.

	FDR < 0.00001			FDR < 0.0001			FDR < 0.001		
Subtotal within the light source									
	Total	Up	Down	Total	Up	Down	Total	Up	Down
Red	2,575	1,004	1,571	3,266	1,295	1,971	4,257	1,752	2,505
Blue	935	352	583	1,342	524	818	1,968	786	1,182
Red/Blue	501	171	330	789	280	509	1,247	484	763
Fluorescent	2,419	808	1,611	3,113	1,047	2,066	4,097	1,447	2,650
Numbers of DEGs mutually detected under one, two, three or four light source conditions									
One	1,506			1,851			2,336		
Two	1,178			1,414			1,697		
Three	468			697			997		
Four	291			435			712		

**Fig 4:**
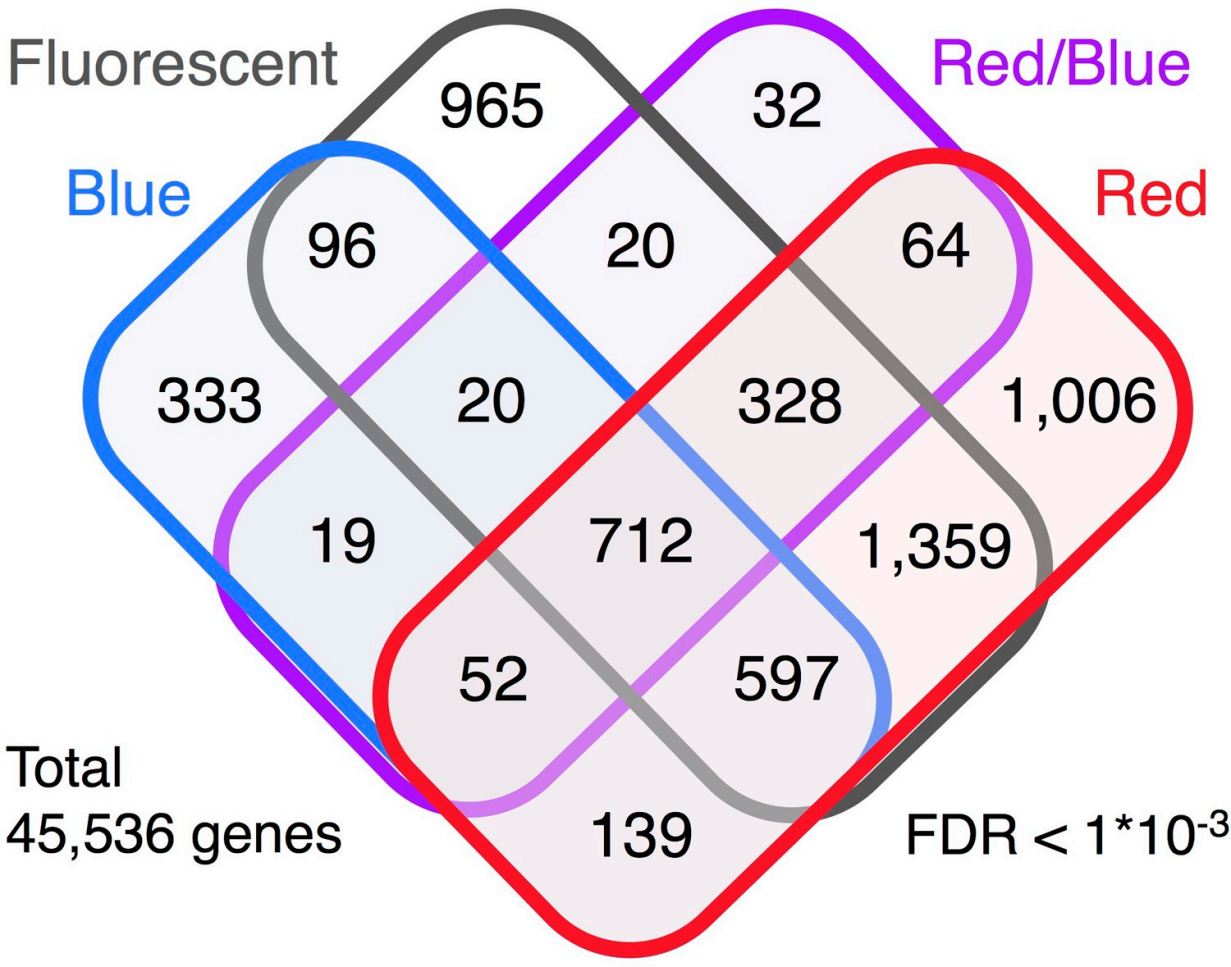
Four-set Venn diagram of the DEGs. The numbers of DEGs with FDR<1*10^−3^ in comparisons of the young and old leaves of the plants grown under the different light source conditions are shown.

The similarity and specificity of the DEGs are shown on a Venn diagram in Fig 4. A total of 712 genes were differentially expressed common between the young and old leaves grown under the four light conditions (FDR <0.001, S3 Table), although the number decreased if the significance level was tightened to FDR<0.00001 (291, Table 3) or FDR <0.0001 (435, Table 3). The numbers of DEGs between the young and old leaves were as follows: 1,006 for the red LEDs, 333 for the blue LEDs, 32 for the red/blue LEDs, and 965 for the fluorescent light (Fig 4). The greatest portion of the DEGs detected under the blue LEDs and the red/blue LEDs were also detected under the red LEDs and the fluorescent light.

### Leaf senescence and the light source-specific patterns of the transcriptomes

**Fig 5:**
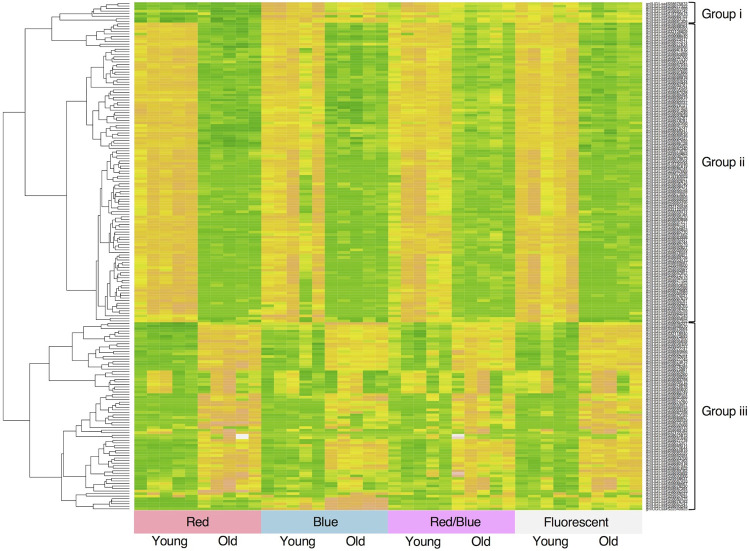
Dendrogram and heat map of the gene expression of the 200 genes with largest variation. The horizontal distance in each branch of the dendrogram represents the degree of similarity between the gene expression profiles of the samples. Each of the columns of the heat map represents an individual. High, intermediate, and low expression levels are shown in pink, yellow, and green colors, respectively. Three groups with the characteristic expression patterns are shown as follows; (i) highly expressed except for the plants grown under the red LEDs, (ii) down-regulated with leaf aging, and (iii) up-regulated with leaf aging.

The expression of the top 200 genes with largest variance are illustrated in a heatmap with categorization in clusters (Fig 5). Three groups showing the following characteristic expression patterns were detected in the heatmap: (i) highly expressed in all plants except those grown under the red LEDs, (ii) down-regulated with leaf aging, and (iii) up-regulated with leaf aging. The first group of eight genes included the following four genes involved in the anthocyanin-synthesis pathway; dihydroflavonol 4-reductase (*DFR*; gnl|UG|Lsa#S58677322), lucoanthocyanidin oxidase (*LDOX*; gnl|UG|Lsa#S56341499), flavanone 3-hydroxylase (*F3H*; gnl|UG|Lsa#S58683142), and transparent testa 4 (*TT4*; gnl|UG|Lsa#S58685102) (S2 Table). A key modulator of cytokinin, *CYCD3*.*2* (gnl|UG|Lsa#S58680279), was observed in the second group (in which the expression level was down-regulated with leaf aging). A gene for leaf senescence-related transcriptional factors, i.e., *WRKY53* (gnl|UG|Lsa#S58685260), was observed in the third group (in which the expression level was up-regulated in the old leaves). Here, *DFR* (gnl|UG|Lsa#S58677322), *LDOX* (gnl|UG|Lsa#S56341499) and *F3H* (gnl|UG|Lsa#S58683142) were reciprocally annotated, and *CYCD3*.*2* (gnl|UG|Lsa#S58680279) and *WRKY53* (gnl|UG|Lsa#S58685260) showed one-side hits with the *A*. *thaliana* database.

### Expression levels of the genes related to leaf senescence and to anthocyanin metabolic pathway

**Fig 6:**
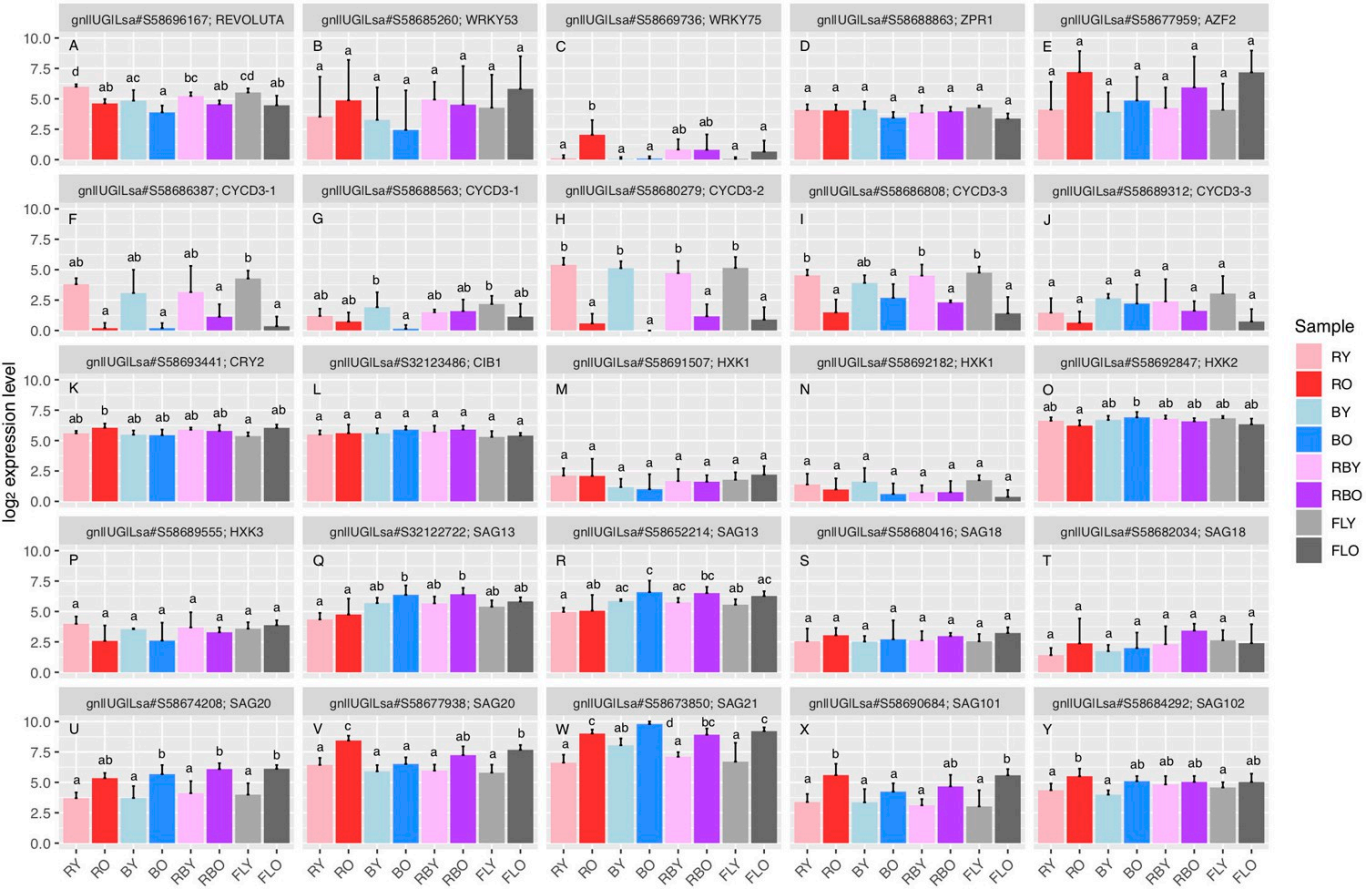
Expression levels of genes involved in leaf senescence. The log_2_ gene expression levels for the leaf samples harvested from young (Y) and old (O) leaves of plants grown under the red (R), blue (B), and red/blue (RB) LEDs and the fluorescent (FL) light are presented on the different bars. Mean ± SD values for five replicate samples are shown. Different superscripts on the bars indicate a significant difference (p<0.05) by ANOVA with Tukey’s HSD test.

To focus on the expressions of genes that may be involved in leaf senescence, we annotated the leaf lettuce genes based on the BLAST search against *A*. *thaliana* genes. The normalized expression levels of 25 of 29 genes recognized as senescence-related are presented in Fig 6; four genes that were not expressed in all of the samples are not shown. Regarding transcriptional regulation factors, young leaves grown under the red LEDs showed significantly higher expression levels of *REVOLUTA* (gnl|UG|Lsa#S58696167) compared to the plants grown under the other light conditions (Fig 6A).

Although there was no significant difference in the expression level of *WRKY53* (gnl|UG|Lsa#S58685260; Fig 6B), the old leaves grown under the red LEDs showed a significantly higher expression level of *WRKY75* (gnl|UG|Lsa#S58669736; Fig 6C), which is a modulator of phosphate acquisition and leaf senescence [35, 36]. There was no significant difference in the expression level of *ZPR1* (gnl|UG|Lsa#S58688863), a target of WRKY53, which is a positive regulator in *A*. *thaliana* [38] (Fig 6D). Although the difference was not significant, the old leaves seemed to have a higher expression level of *AZF2* (gnl|UG|Lsa#S58677959) compared to the young leaves (Fig 6E).

Regarding members of the *CYCD3* gene family (Fig 6F–6J), *CYCD3* is essentially implicated in processes involved in cell division and endoreduplication, which are in part the control of cytokinin, we observed that the young leaves had significantly higher expression levels of *CYCD3-1* (gnl|UG|Lsa#S58686387; Fig 6F), *CYCD3-2* (gnl|UG|Lsa#S58680279; Fig 6H) and *CYCD3-3* (gnl|UG|Lsa#S58686808; Fig 6I) compared to the old leaves. For the blue light-responsive genes related to leaf senescence, we observed that the old leaves grown under the red LEDs had significantly higher expression levels of *CRY2* (gnl|UG|Lsa#S58693441; Fig 6K), and there was no significant difference among the light conditions and leaf ages in *CIB1* (gnl|UG|Lsa#S32123486; Fig 6L). The genes that encode hexokinases (HXKs), which interact with the senescence-regulatory pathway and act as glucose sensors, i.e., *HXK1* (gnl|UG|Lsa#S58691507 and gnl|UG|Lsa#S58692182; Fig 6M and 6N) and *HXK3* (gnl|UG|Lsa#S58689555; Fig 6P), did not show a significant difference among the light conditions. However, *HXK2* (gnl|UG|Lsa#S58692847) tended to be suppressed in the old leaves of the plants grown under the red LEDs (Fig 6O).

There were three types of response for *SAGs* with leaf aging. Firstly, regarding *SAG13* (gnl|UG|Lsa#S32122722 and gnl|UG|Lsa#S58652214; Fig 6Q and 6R), the leaves grown under the red LEDs had lower expression levels compared to the leaves grown under the light containing blue wavelength. Secondly, for *SAG20* (gnl|UG|Lsa#S58674208 and gnl|UG|Lsa#S58677938; Fig 6U and 6V), *SAG21* (gnl|UG|Lsa#S58673850; Fig 6W), and *SAG101* (gnl|UG|Lsa#S58690684; Fig 6X), the expression levels increased with leaf aging, except for the leaves exposed to blue light, in which the expression levels of the two genes (*SAG20*; gnl|UG|Lsa#S58677938; Fig 6V and *SAG101*; gnl|UG|Lsa#S58690684; Fig 6X) did not change significantly. Thirdly, the old leaves in the plants grown under the red LEDs showed significantly higher expression levels of *SAG102* (gnl|UG|Lsa#S58684292) compared to the young leaves of the plants grown under the red LEDs, blue LEDs, and fluorescent light tubes (Fig 6Y). *SAG18* (gnl|UG|Lsa#S58680416 and gnl|UG|Lsa#S58682034; Fig 6S and 6T) did not show a significant difference among the four light conditions (Fig 6S and 6T). The same trends were observed for *WRKY75* (gnl|UG|Lsa#S58669736) with the real-time RT-PCR (S5 Fig), although large experimental variances were detected between different samples. Here, *REVOLUTA* (gnl|UG|Lsa#S58696167), *WRKY75* (gnl|UG|Lsa#S58669736), *AZF2* (gnl|UG|Lsa#S58677959), *HXK2* (gnl|UG|Lsa#S58692847), *HXK3* (gnl|UG|Lsa#S58689555), *SAG18* (gnl|UG|Lsa#S58682034), *SAG20* (gnl|UG|Lsa#S58677938), and *SAG101* (gnl|UG|Lsa#S58690684) were reciprocally annotated, and other genes showed one-side hits in the BLAST search with the *A*. *thaliana* database.

The expression patterns of the genes related to the anthocyanin biosynthesis pathway are shown in S3 Fig. A total of 73 genes were recognized as related to the anthocyanin synthesis pathway. In each reaction step, several homologous genes were identified (S3 Fig), which indicated that there is a comparable anthocyanin synthesis pathway with *A*. *thaliana* (S3 Fig). Through the pathway, there were suppressions in major expressed genes in *CHS* (gnl|UG|Lsa#S58685102), *DFR* (gnl|UG|Lsa#S58677322), and *LDOX* (gnl|UG|Lsa#S56341499) in the plants grown under the red LEDs compared to the plants grown under the other light conditions (p<0.05 by ANOVA, S3 Fig). In addition, the gene expression levels of *C4H* (gnl|UG|Lsa#S58689833 and gnl|UG|Lsa#S58689971), *CHI* (gnl|UG|Lsa#S58678838 and gnl|UG|Lsa#S58678730), and *FLS* (gnl|UG|Lsa#S58681956) were decreased with leaf aging (S3 Fig). The same trends were observed for *PAL* (gnl|UG|Lsa#S18832770) and *CHS* (gnl|UG|Lsa#S58685102) with the real-time RT-PCR (S5 Fig). Here, *DFR* (gnl|UG|Lsa#S58677322), *LDOX* (gnl|UG|Lsa#S56341499), *C4H* (gnl|UG|Lsa#S58689971), *CHI* (gnl|UG|Lsa#S58678838 and gnl|UG|Lsa#S58678730), and *FLS* (gnl|UG|Lsa#S58681956) were reciprocally annotated, and other genes showed one-side hits in the BLAST search with the *A*. *thaliana* database.

Our GO enrichment analysis performed for the DEGs detected by the exact test in the comparison of the young leaves grown under the red LEDs and the blue LEDs demonstrated that the number of upregulated genes and their GO annotations under the red LEDs were relatively smaller than those under the blue LEDs (S4 Fig). The Biological Process results containing the terms “response to stress” and “response to abiotic stimulus” were greater under the blue LEDs compared to the red LEDs (S4 Fig). In the Biological Process results, the “metabolic process” connecting to “primary metabolic process” and “carbohydrate metabolic process” terms were significantly higher among the genes that were upregulated under the blue LEDs. In the Cellular Component and Molecular Function results, there were also large numbers of genes that were overexpressed under the blue LEDs.

## Discussion

Our profiling based on the RNA-Seq revealed a clear difference in transcriptomes between young and old leaves, and we observed that red LED light caused large differences between the two age classes, whereas the pure and mixed blue LED light induced smaller differences in transcriptomes between the young and old leaves (Figs 3 and 4). Both under the red LEDs and under the fluorescent light, which are conditions that increase the plant mass, there was a large difference between transcriptomes of the young and old leaves of the plants. These results suggest that the differences in the growth-light quality and the difference in leaf age were clearly reflected in its transcriptomes.

Red and blue lights affect the control of plant growth and development through specific light photoreceptors. Three major families of photoreceptors have been identified and characterized in higher plants: red/far-red light-absorbing phytochromes, and blue/UV-A light-absorbing cryptochromes [44] and phototropins [45]. These families of photoreceptors mediate a number of similar physiological responses [46]. Although there are several points of convergence between phytochrome and cryptochrome signaling [46–48] and although co-action between phytochromes and cryptochromes has been reported [49], the two cascades after red- and blue-light photoreception can be distinguished from each other. In the ordinary red-light response through phytochromes, the balance between red-light and far-red light is important in controlling the downstream reactions, because radiation in the far-red region is poorly absorbed by leaves, and thus the light that is transmitted through or reflected from vegetation is depleted in red and significantly enriched in far-red wavelengths [50]. In response to a low ratio of photon irradiance in the red to that in the far-red (R:FR ratio) signals, plants display a rapid and pronounced increase in the elongation growth rate of stems and petioles, often at the expense of leaf and storage organ development [50]. Leaves recognize their own light environment with an R:FR ratio, and control leaf senescence. An irradiation with red light suppressed the degradation of chlorophyll during subsequent dark periods and the effect of red light was nullified by an irradiation with far-red light in rice leaves [51]. These physiological findings suggest that the initiation of leaf senescence is mediated through the sensing of the low R:FR ratio by phytochromes in order to use resources efficiently and reallocate this into novel modules in plants.

However, R:FR ratio was sustainably maintained at a high level under the artificial irradiance of the red LED light (S1 Fig). Light with a high R:FR ratio does not cause a leaf to sense that it lies under another leaf, and such light is not a signal for the onset of leaf senescence. In addition, red LED light does not stimulate leeward cascades of the blue-light absorbing photoreceptors, cryptochromes and phototropins. On the other hand, we observed that an influence of the dwarfing of the leaves promoted by blue light [52] affected the fresh and dry mass values of the aerial part of the plants grown under the blue and red/blue LEDs. Genes involved in environmental stress were upregulated under the blue LEDs compared to their levels under the red LEDs (S4 Fig), suggesting that the plants prioritized their stress responses over their growth under the blue LEDs.

Such a biased photoresponse due to an uneven light wavelength was remarkable in the behavior of the individual gene expression patterns. In this study, the most highly expressed gene gnl|UG|Lsa#S58677322 in dihydroflavonol 4-reductase (*DFR*) coding genes and gnl|UG|Lsa#S56341499 in lucoanthocyanidin oxidase (*LDOX*) coding genes, which are the key enzymes of the anthocyanin-synthesis pathway, were not well expressed in the plants grown under the red LEDs (Fig 5 and S3 Fig), although they were highly expressed under the blue LED and fluorescent light tubes (Fig 5 and S3 Fig). It is known that lettuce leaves do not accumulate anthocyanins when growing under red light but accumulate a large amount of anthocyanins when growing under blue light [52], and the same trend was observed in this study (Fig 1).

In addition, genes that indicate the status of leaf senescence provide details about the physiological status of the leaves. The expressions of *CYCD3-1* (gnl|UG|Lsa#S58686387), *CYCD3-2* (gnl|UG|Lsa#S58680279), and *CYCD3-3* (gnl|UG|Lsa#S58686808) were significantly higher in the young leaves than in the old leaves in the plants grown under the red, blue, red/blue LEDs, and fluorescent light tubes (Fig 6F, 6H and 6I). Members of the CYCD3 family have functional roles to coordinate hormonal responsibility with cytokines and are usually expressed in the immature tissues of leaves [37]. SAG family genes that are involved in leaf senescence tended to be associated with leaf aging with inherent patterns in the present study (Fig 6Q–6Y). The genes whose expression increased with leaf senescence (e.g., SAG13) were suppressed under red LED, suggesting that red LEDs with a high R:FR ratio do not cause a leaf to sense that it lies under another leaf, and such light is not a signal for the onset of leaf senescence. The low wastage rate for lettuce plants grown under red LEDs [7] could be related to senescence-suppression upstream factors, e.g., *REVOLTA* (Fig 6A), and downstream senescence genes, e.g., SAG family genes. In contrast, the anti-aging effect of red light may be partially counteracted under fluorescent light, including far-red light, since the expressions of senescence-associated genes (e.g., SAG13 and SAG20) were higher under fluorescent light. Differences in the expression patterns of individual genes clearly reflect the differences in the physiological status of the lettuce plants caused by the growth light condition as well as leaf aging.

Since the detailed physiological conditions of leaves can be monitored by consulting the gene expression profiles, transcriptome data provide useful information for lettuce cultivation management. Here, the transcriptome showed differences that do not appear in traits, such as an increase in the biomass due to delayed senescence and the nutritional deficiency of the plants grown under the red LEDs. Using the expression levels of characteristic genes as an index to physiological conditions will make it possible to devise better growing environments in plant factories based on the clear trends of the marker genes. For example, the high expression of *WRKY75* (a transcriptional factor expressed under phosphate deprivation in *A*. *thaliana*; Fig 6C) in the old leaves grown under the red LEDs herein suggests that a further extension of the leaf lifespan and an increase in the individual plant weights occur when a phosphorus deficiency is eliminated. Previous studies have shown that red-light induces the activation of phosphate uptake [53], and the phosphorus requirement of plants is higher under red light [54]. It may be possible to reduce plant wastage by identifying the conditions under which the aging-related genes are suppressed. In the present study, we observed that the lettuce plants grew better (considering the number of leaves, total biomass, and the shoot/root ratio) under fluorescent light than under most of the LED light conditions. This indicates that the efficiency of cultivation per unit of light intensity may be higher under fluorescent light than under other biased light wavelengths. Fluorescent light has several peaks, and some of them are close to the PSI and PSII absorption maxima in photochemical reactions. In this sense, the extent to which the switch from fluorescent light to LEDs during the seedling growth stage and the plant growth stages had an effect on growth merits further consideration. Notably, plants in the germination and seedling growth stages were grown under the same fluorescent light to reduce variability before application of the different light treatments in this study, and seedling growth in actual commercial plant factories is conducted under fluorescent lights. Because green light can penetrate further into a leaf than red or blue light, any additional green light absorbed by the lower chloroplasts increases the leaf photosynthesis to a greater extent than additional red or blue light, under strong light conditions [55]. Although green light has been suggested to balance the biomass production with the production of secondary metabolites in lettuce [27], the effect of green light on leaf senescence needs to be investigated in a future study.

Since the expression levels of genes related to the anthocyanin synthesis pathway were consistent with the accumulation pattern of anthocyanin according to the growth light conditions [52], information about the gene expression levels will be useful in attempts to regulate the accumulation of anthocyanins by adjusting the light conditions and to obtain the desired quality. Gene expressional levels can thus be considered expression markers for establishing or monitoring optimal or suboptimal conditions in order to achieve better production in plant factories. Although further research is necessary to evaluate the effects of improvements based on modifications of growth conditions, the results of this study suggest that gene expression profiling and the resulting knowledge of functional genes can be an important tool to improve plant cultivation methods. While massive gene profiling *per se* does not seem cost-effective, it may help researchers to identify important genes, which could then be used as markers to improve cultivation methods under specific environmental conditions.

## Supporting information

S1 Fig Spectral characteristics of the red LEDs, blue LEDs, and the fluorescent light sources. (TIF)

S2 Fig Hierarchical clustering of transcriptomes in the plants grown under different light source conditions. (TIF)

S3 Fig Expression levels of genes in the anthocyanin synthesis pathway. (TIF)

S4 Fig GO pathways for the DEGs detected by the exact test in young leaves grown under red LEDs and blue LEDs light conditions. (TIF)

S5 Fig Box plots of the relative expression levels of genes related to leaf senescence and anthocyanin contents with real-time RT-PCR. (TIF)

S1 Table Summary statistics of the transcriptome analysis. (XLSX)

S2 Table The 200 genes with the largest variations in expression. (XLSX)

S3 Table Commonly detected genes in the comparisons of the young and old leaves through the four light conditions. (XLSX)

S4 Table Summary of the gene expression analysis with real-time RT-PCR. (XLSX)
