# Supplementary figures and images for: Effect of differences in light source environment on transcriptome of leaf lettuce (Lactuca sativa L.) to optimize cultivation conditions
Source: PLoS One. 2022 Mar 29;17(3):e0265994. doi: 10.1371/journal.pone.0265994 (PMC8963549; doi:10.1371/journal.pone.0265994)

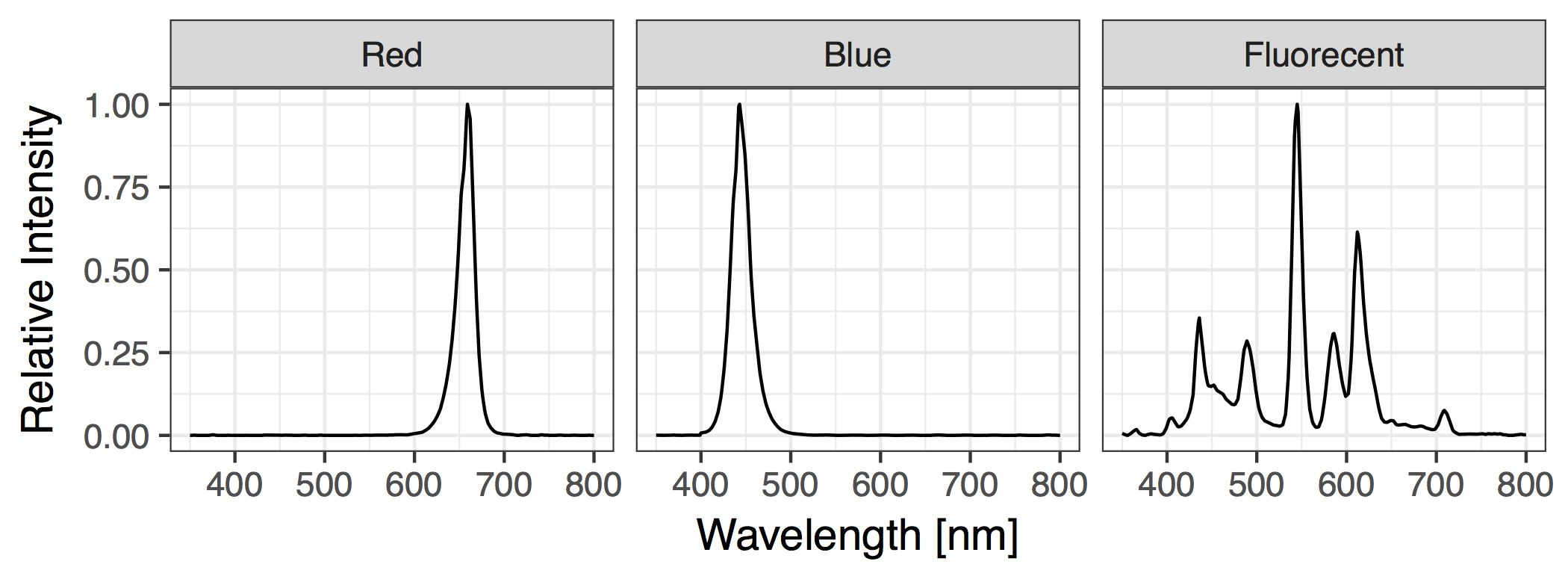

Supplement: S1 Fig — (TIF) [file pone.0265994.s001.tif]

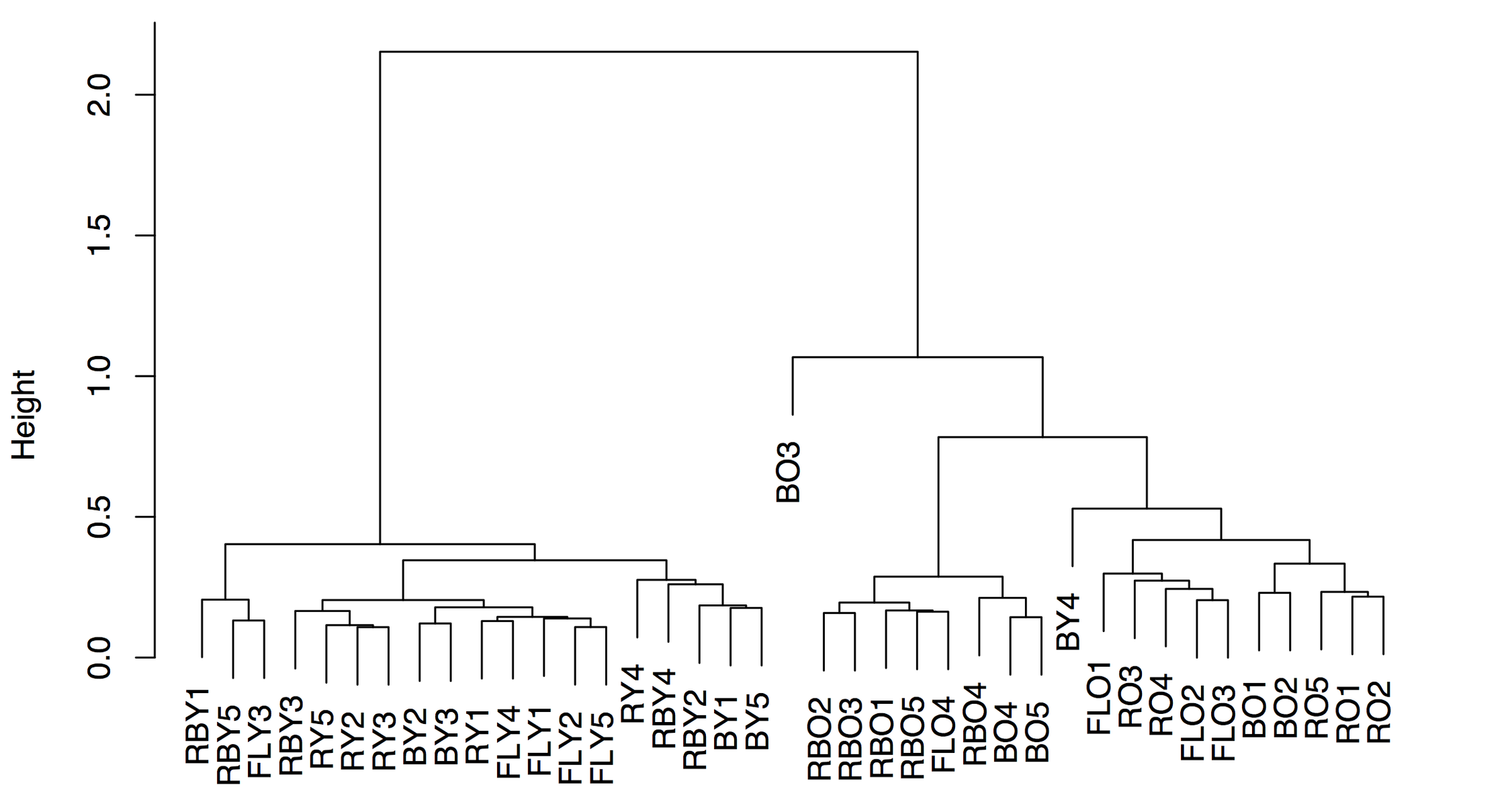

Supplement: S2 Fig — (TIF) [file pone.0265994.s002.tif]

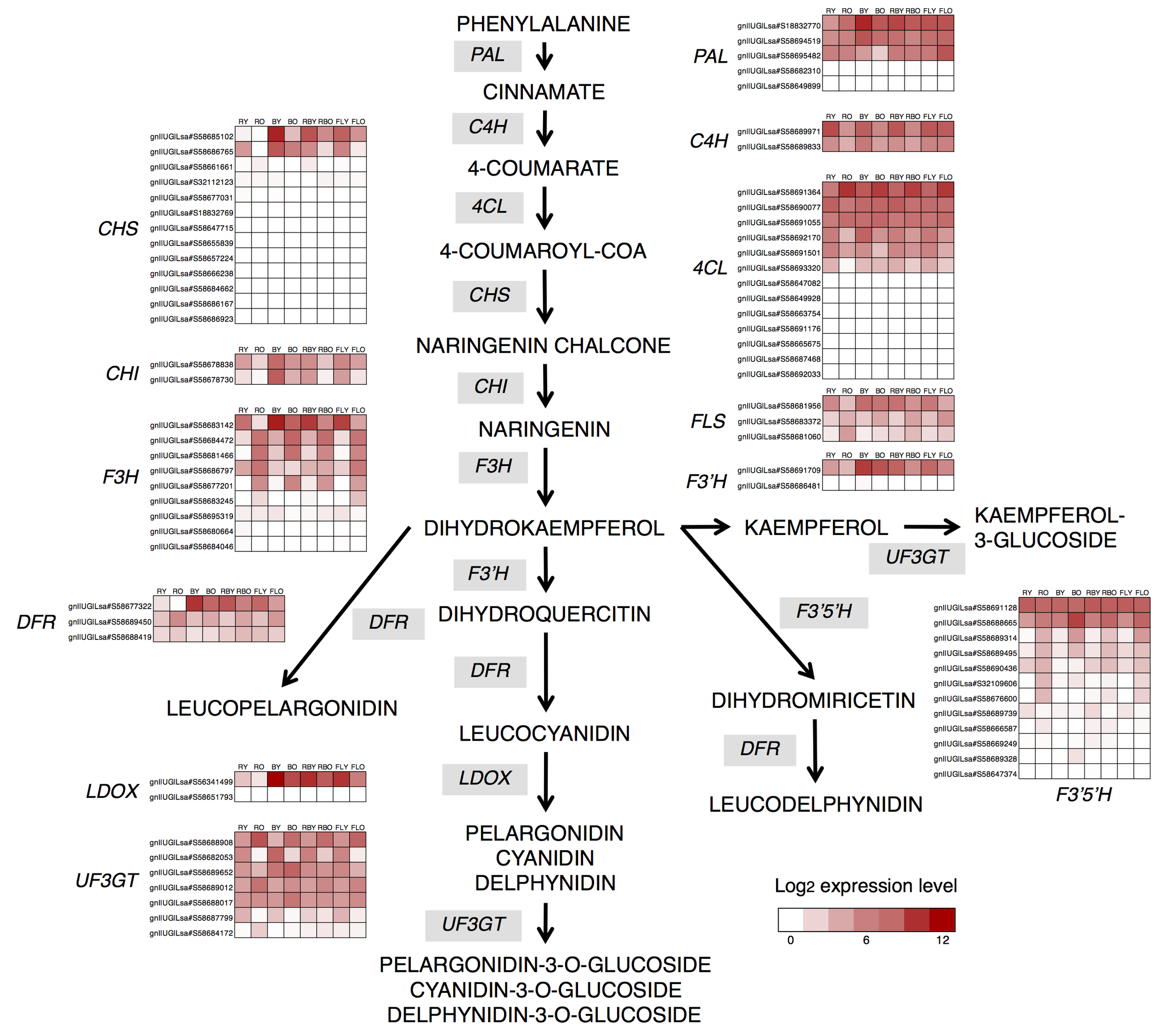

Supplement: S3 Fig — (TIF) [file pone.0265994.s003.tif]

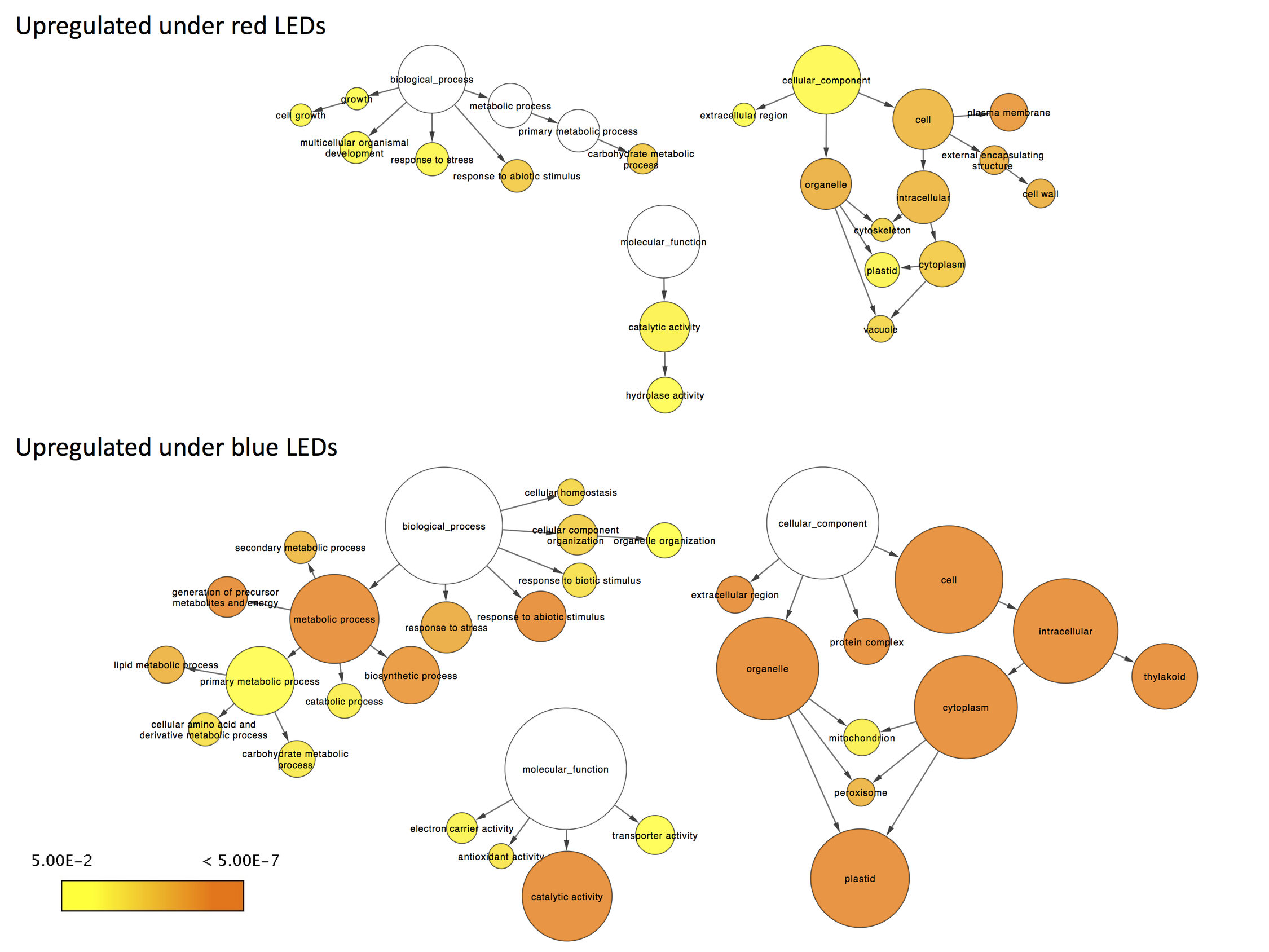

Supplement: S4 Fig — (TIF) [file pone.0265994.s004.tif]

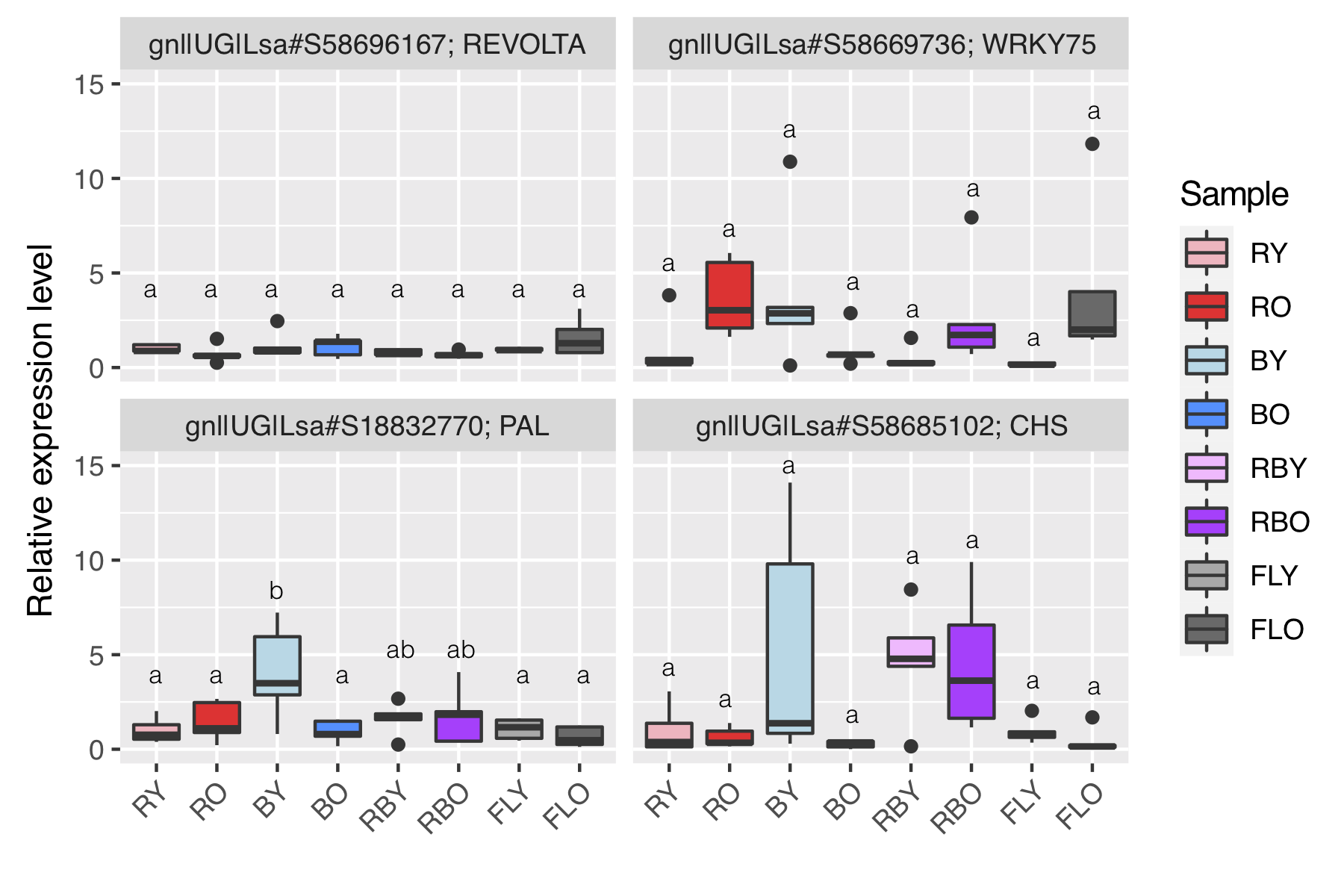

Supplement: S5 Fig — (TIF) [file pone.0265994.s005.tif]
